# The role of ADAMTS‐13 activity and complement mutational analysis in differentiating acute thrombotic microangiopathies

**DOI:** 10.1111/jth.13189

**Published:** 2016-01-11

**Authors:** E. H. Phillips, J. P. Westwood, V. Brocklebank, E. K. S. Wong, J. O. Tellez, K. J. Marchbank, S. McGuckin, D. P. Gale, J. Connolly, T. H. J. Goodship, D. Kavanagh, M. A. Scully

**Affiliations:** ^1^ Department of HaematologyUniversity College LondonLondonUK; ^2^ Institute of Genetic MedicineNewcastle UniversityNewcastle upon TyneUK; ^3^ Institute of Cellular MedicineNewcastle UniversityNewcastle upon TyneUK; ^4^ Centre for NephrologyUniversity College LondonLondonUK; ^5^ Department of NephrologyRoyal Free HospitalLondonUK; ^6^ Cardiometabolic ProgrammeNIHR/University College London Hospitals Biomedical Research CentreLondonUK

**Keywords:** atypical hemolytic uremic syndrome, CD46, complement, diagnosis, thrombotic thrombocytopenic purpura

## Abstract

**Essentials**

- Molecular diagnostics has improved the differentiation of acute thrombotic microangiopathys (TMAs).
- Atypical hemolytic uremic syndrome may have features mimicking thrombotic thrombocytopenic purpura.
- We identified novel complement mutations and a high incidence of *CD46*, with favorable long term outcomes.
- Complement mutation analysis in TMA where the diagnosis is unclear and ADAMTS‐13 activity is >10%.

**Summary:**

**Background**

Differentiation of acute thrombotic microangiopathy (TMA) at presentation has historically been dependent on clinical parameters. Confirmation of thrombotic thrombocytopenic purpura (TTP) is increasingly reliant on demonstrating deficient ADAMTS‐13 activity. The identification of alternative complement pathway abnormalities in atypical hemolytic uremic syndrome (aHUS), along with the proven efficacy of terminal complement inhibitors in treatment, has increased the need for rapid differentiation of TTP from aHUS.

**Objectives**

We describe the clinical phenotype and nature of complement mutations in a cohort of aHUS patients referred as acute TMAs.

**Patients/methods**

Fourteen consecutive aHUS patients were screened for mutations in *C3*,*CD46*,*CFH*,*CFI*, and *CFB*, as well as factor H (FH) antibodies. All aHUS patients had ADAMTS‐13 activity > 10%.

**Results**

Of 14 aHUS patients, 11 (79%) had platelet counts < 30 × 10^9^/L during the acute phase. Median presenting creatinine level was 295 μmol L^−1^, while five (36%) of 14 presented with a serum creatinine level < 200 μmol L^−1^. Alternative complement pathway mutations were detected in 9 (64%) of 14 patients, including *CD46* mutations in five (36%) of 14 patients. Patients were identified with novel mutations in *CFB* and *C3* that have not been previously reported.

**Conclusions**

We demonstrate that diagnostic differentiation based on platelet count and renal function is insufficient to predict an underlying complement mutation in some aHUS cases. Specifically, we demonstrate a high frequency of functionally significant *CD46* mutations which may mimic TTP. ADAMTS‐13 activity > 10% in a patient with a TMA should necessitate genetic screening for complement abnormalities.

## Introduction

Atypical hemolytic uremic syndrome (aHUS) and thrombotic thrombocytopenic purpura (TTP) are thrombotic microangiopathies (TMA) that manifest with organ dysfunction or life threatening features requiring urgent recognition and treatment. Both present with microangiopathic hemolytic anemia and thrombocytopenia, although the clinical course of the two diseases can be markedly different. Historically, ~60% of aHUS patients progressed to end‐stage renal failure (ESRF) 1, 2, whereas in TTP, neurological and cardiac features are often more prominent and severe renal dysfunction is rare.

Although it is known that aHUS and TTP are histologically distinct entities 3, diagnosis is often reliant on clinical features, such as the pattern of organ dysfunction and degree of thrombocytopenia. It has been suggested that a serum creatinine level of > 150 to 200 μmol L^−1^ or a platelet count of > 30 × 10^9^ L^−1^ ‘almost eliminates’ a diagnosis of TTP 4. Because the two diseases have overlapping clinical features, however, there is inherent opportunity for misdiagnosis, and studies on TTP and aHUS have been open to diagnostic bias. The term ‘TTP‐HUS’ has been used historically to reflect the degree of diagnostic uncertainty.

The past decade has seen major advances in our knowledge of the molecular pathophysiology underlying both aHUS and TTP, accompanied by shifts in diagnostic testing and divergence of treatment algorithms for the two diseases. In aHUS, dysregulation of the alternative complement pathway leads to excessive activation of the terminal complement pathway, complement‐mediated endothelial cell damage, and subsequent glomerular microthrombi 2. Detectable complement abnormalities have been described in ~50% of patients, including loss of function mutations within membrane co‐factor protein (*CD46*), complement factor H (*CFH*) and factor I (*CFI*), and autoantibodies to the factor H (FH) and factor I (FI) proteins. Gain‐of‐function mutations have also been identified within complement factor B (*CFB*) and *C3* 5, 6, 7.

Thrombotic thrombocytopenic purpura, caused by an acquired or inherited deficiency of the von Willebrand factor–cleaving protease ADAMTS‐13 (a disintegrin and metalloproteinase with a thrombospondin type 1 motif member 13), results in spontaneous aggregation of platelets and ultralarge von Willebrand multimers to form platelet‐rich thrombi throughout the microvasculature 8. Assessment of ADAMTS‐13 activity is now routinely performed in patients presenting with acute TMA and, when < 10%, aids in the differentiation of TTP from other causes of TMA 9, 10.

Early initiation of either immunomodulatory therapy in TTP 11 or complement blockade in aHUS 12 can improve outcomes, increasing the need for prompt and accurate differentiation between the TMAs.

We describe a retrospective cohort of aHUS patients referred with acute TMA and clinically suspected TTP. We highlight their presenting clinical and laboratory features, and subsequent complement abnormalities. This study emphasizes the clinical overlap and pivotal role of ADAMTS‐13 assessment in the differentiation of TMAs. We also outline response to therapy and long‐term outcomes of this aHUS subgroup, before complement inhibitors were available.

## Patients and methods

Data were retrospectively analyzed for all patients with a final diagnosis of aHUS managed at University College Hospital London between January 2006 and July 2013. Twelve patients were transferred during an acute presentation with a presumptive diagnosis of TTP based on clinical parameters. Two patients were referred to TTP clinic following prior episodes of TMA to exclude a diagnosis of congenital TTP (patients 1 and 6, Tables 1 and 2). All patients had evidence of TMA, defined as the presence of thrombocytopenia with microangiopathic hemolytic anemia (anemia, schistocytes on blood film, reticulocytosis, hyperbilirubinemia, elevated lactate dehydrogenase, and negative direct antiglobulin test) 13, during their initial presentation. Citrated blood for ADAMTS‐13 levels was taken prior to therapeutic plasma exchange (TPE). Patients were also tested for human immunodeficiency virus, antinuclear antibodies (ANA), lupus anticoagulant, human chorionic gonadotropin, and fecal O157 enterotoxin, if reporting diarrheal symptoms, on presentation. C3 and C4 levels were measured prior to TPE. Patients with secondary TMA, including transplant‐associated microangiopathy, active rheumatologic disease, malignancy, and drug‐induced TMA, were excluded.

**Table 1:** Clinical features and presenting laboratory features of aHUS patients

Patient ID	Gender	Age (years)	Familial?	Triggering events^a^	ADAMTS‐13 activity (%)	Platelet count (×10^9^/L)	Creatinine (μmol L^−1^)	Hb (g L^−1^)	LDH (IU L^−1^)	Total bilirubin (μmol L^−1^)	Objective neurological involvement	Dialysis	Outcome	Recurrences
1	F	0.92	No	1, 2, 3	107	11	290	88	H	54	No	Yes	CR	4
2	M	21	No	1	86	20	443	114	2083	26	No	Yes	CR	0
3	M	15	Yes	1	93	15	94	120	1986	40	No	No	CR	1
4	M	3	Yes	1	96	18	79	102	1066	38	No	No	CR	1
5	M	44	No	1	78	10	357	122	771	16	No	No	CR	0
6	F	28	No	0	88	115	1812	85	2162	32	No	Yes	Stage 3 CKD	0
7	F	35	No	4	77	26	212	58	3604	20	No	No	CR	0
8	F	23	No	1, 3	86	28	260	93	4621	51	TIA	No	CR	1
9	M	72	No	0	84	56	194	95	541	23	TIA	No	CR	1
10	F	36	No	4	50	16	672	66	2047	32	No	No	CR	0
11	M	36	No	0	72	34	137	89	2114	67	No	No	CR	0
12	F	23	No	2	48	31	608	105	342	63	No	No	CR	0
13	F	20	No	2	47	28	300	94	581	77	No	No	CR	0
14	F	51	No	0	120	58	198	72	H	81	No	No	Death	0

a Triggering events at initial presentation ± relapse: 0 = none identified, 1 = upper respiratory tract infection, 2 = abdominal sepsis, 3 = vaccination, 4 = pregnancy. LDH: H, hemolyzed. Outcome: CR, complete remission, i.e., normaliation of creatinine and platelet count. CKD, chronic kidney disease; TIA, transient ischemic attack.

**Table 2:** Summary of complement analysis of aHUS patients with ADAMTS‐13 activity > 10%

Patient ID	Gene	Mutation identified	C3 (0.65–1.65 g L^−1^)	C4 (0.16–0.54 g L^−1^)	FH (0.35–0.59 g L^−1^)	FI (38–58 mg L^−1^)	Copies *CFHR1/3*	*CD46*GGAAC haplotype copies	*CFH* H3 haplotype copies	Mutation reported previously
1	*CD46*	c.286+2T>C c.286+2T>G*	0.65†	0.14	0.5	39	2	1	0	7, 20, 28, 45
2	*CD46*	c.175C>T; p.Arg59X	0.56	0.04	0.5	49	1	2	0	27, 28, 46
3	*CD46*	c.470G>A;p.Cys157Tyr	0.92†	0.26	0.51	44	2	1	2	
4	*CD46*	c.470G>A;p.Cys157Tyr	1.31	0.27	0.58	62	2	1	2	
5	*CD46*	c.191G>T; p.Cys64Phe	1.17	0.17	0.78	84	2	1	0	29
6	*CFH*	c.3134‐5T>C	0.87	0.23	0.62	53	2	0	2	
7	*CFH*	c.3643C>T p.Arg1215X	0.85	0.31	0.42	73	2	2	1	1
8	*CFB*	c.1112A>G p.Asp371Gly	0.91	0.3	0.67	77	1	1	0	
9	*C3*	c.3023C>T; p.Ser1008Leu	1.15†	0.34	0.62	60	2	1	1	
10	Nil	–	1.46	0.31	0.52	66	0	1	0	
11	Nil	–	0.56	0.03	0.55	52	2	0	2	
12	Nil	–	0.4	0.09	–	–	2	2	1	
13	Nil	–	0.94	0.24	0.62	50	1	2	0	
14	Nil	–	1.3	0.32	0.74	77	2	1	1	

*Compound heterozygote. †Complement levels measured on convalescent samples. The number of risk alleles for the *CFH*‐H3 haplotype block that increases the risk of aHUS two‐ to four‐fold 1, 47 and the *CD46* GGAAC haplotype block that has been associated with a two‐ to three‐fold increased risk of aHUS 1, 48 are shown. Complete deficiency of *CFHR1* and *CFHR3* has been strongly associated with factor H autoantibodies and aHUS 49, 50, 51, 52. Only patient 10 carried this deletion in homozygosity; however, no factor H autoantibodies were detected in this study.

Atypical hemolytic uremic syndrome was diagnosed according to criteria published by the UK aHUS Rare Diseases Group 14 and European guidelines 15, including the presence of both TMA and acute kidney injury 16 without ADAMTS‐13 deficiency or inhibitors. One patient underwent renal biopsy for diagnostic confirmation. Two patients did not fit diagnostic criteria due to normal renal function but were investigated due to recurrent TMA with normal ADAMTS‐13 activity; the identification of a functionally significant mutation in one of the aforementioned complement genes was supportive of a diagnosis of aHUS in both patients. All analyses, including mutation screening, were undertaken as part of routine patient care.

We assessed whether coexisting complement mutations were present in TTP cases and could account for increased disease severity or renal impairment. Mutation screening was undertaken in 14 TTP patients (ADAMTS‐13 activity < 10% and detectable anti‐ADAMTS‐13 IgG auto antibodies) with either renal impairment or a severe phenotypic presentation. Sample use was approved by the local research ethics committee (reference 08/H0716/72).

### ADAMTS‐13 assays

ADAMTS‐13 activity was measured by fluorescence resonance energy transfer–von Willebrand factor 73 17. An ADAMTS‐13 level < 10% (normal range: 60–123%) was confirmatory of a diagnosis of TTP in all selected patients. Patients were screened for acquired IgG inhibitors as previously described 18, with a normal range of < 6.1% calculated as the 95th percentile of 49 normal healthy controls.

### Complement assays

C3 and C4 levels were measured by rate nephelometry (Beckman Coulter Array 360, Ramsey, MN, USA). FH and FI levels were measured by radioimmunodiffusion (Binding Site, Birmingham, UK), Screening for FH autoantibodies was undertaken using ELISA as described previously 19. FACS analysis of granulocytes from the patients was performed as described previously 20.

### Genetic analysis

Mutation screening of *CFH* 21, *CFI* 22, *CFB* 23, *CD46* 24, and *C3* 25 was undertaken using Sanger sequencing as previously described. Genotyping of the following SNPs *CFH* −331C>T (rs3753394), *CFH* c.184G>A; p.Val62Ile (rs800292), *CFH* c.1204T>C; p.Tyr402His (rs1061170), *CFH* c.2016A>G; p.Gln672Gln (rs3753396), *CFH* IVS15 −543G>A intron 15 (rs1410996), *CFH* c.2808G>T; p.Glu936Asp (rs1065489), *CD46* −652A>G (rs2796267), *CD46* −366A>G (rs2796268), *CD46* IVS9 −78G>A (rs1962149), *CD46* IVS12 +638G>A (rs859705), and *CD46* c.4070T>C (rs7144) was used to determine *CFH* and *CD46* haplotypes 26.

### Multiplex ligation–dependent probe amplification

Screening for genomic disorders affecting *CFH*,* CFHR1*,* CFHR2*,* CFHR3*, and *CFHR5* was undertaken using multiplex ligation–dependent probe amplification 26.

### Analysis of *CFH* c.3134‐5T>C variant

RNA was extracted from peripheral blood using RNAeasy Mini kit (Qiagen, Manchester, UK). cDNA was synthesized with SuperScript III First‐Strand Synthesis System (Invitrogen, Thermo Fisher Scientific, Paisley, UK) using random hexamers and the extracted RNA as a template. cDNA was used as a template in a polymerase chain reaction with specific primers targeting the potential splice‐site mutation (*CFH* exon 20 [F‐TATAA GGCGGGTGAGCAAGT] and *CFH* exon 23[AACTGATTCACCTGTTCTCG]). Polymerase chain reaction products were separated on a 2% TBE agarose gel and sequenced using ABI Big Dye Terminator v3.1 on an ABI 3500 Genetic Analyzer (Life Technologies, Thermo Fisher Scientific).

### Western blotting

Detection of potential abnormal protein products in serum arising as a consequence of the *CFH* c.3134‐5T>C variant was undertaken by Western blotting. Sera was diluted 1:1500, and 10 μL was electrophoresed on 6% sodium dodecyl sulfate polyacrylamide gel electrophoresis gel and transferred onto nitrocellulose. A polyclonal antibody against FH (Calbiochem, Beeston, Nottingham, UK) was used with rabbit anti‐goat horseradish peroxidase (Abcam, Cambridge, UK). Following washes in Tris‐buffered saline with Tween 20, the blot was developed using Pierce ECL Western blotting substrate (Thermo Scientific).

## Results

### aHUS cases

**Figure 1:**
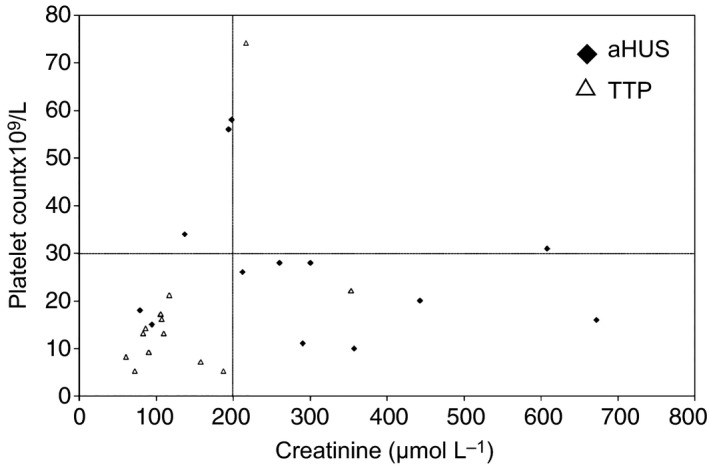
Serum creatinine and platelet values at presentation in thrombotic thrombocytopenic purpura (TTP) and atypical hemolytic uremic syndrome (aHUS) patients. Previously suggested cut‐off levels for platelet count (30 × 10^9^ L^−1^) and creatinine of 200 μmol L^−1^ in aHUS 4 are highlighted. One aHUS patient, who presented with a platelet count of 115 × 10^9^ ^1^/L and creatinine of 1812 μmol L^−1^, is censored. Two TTP patients presented with identical platelet counts and serum creatinine levels.

Clinical features and laboratory parameters at presentation are detailed in Table 1. Median age at first episode was 25.5 years (9 months to 72 years), and 43% were male. The median presenting platelet count was 27 × 10^9^ L^−1^ (range 10–115), serum creatinine level was 295 μmol L^−1^ (79–1812), and ADAMTS‐13 activity was 80.8% (47–120%). Eleven (79%) of 14 patients had platelet counts < 30 × 10^9^ L^−1^ during the acute phase (nine of 14 at presentation; Fig. 1). Although renal involvement was more common and more severe in the group subsequently diagnosed to have aHUS, 5 (36%) these 14 patients had a serum creatinine level < 200 μmol L^−1^ on transfer to our center; in two patients, serum creatinine was entirely within normal limits (Table 1, patients 3 and 4). Three (21%) of 14 maintained a creatinine < 200 μmol L^−1^ throughout the acute episode. Only one patient had a non‐specific weakly positive ANA at a titer of 1:80 (patient 6). All aHUS patients had ADAMTS‐13 activity levels > 30% (median 85%, range 47–120%) and no detectable ADAMTS‐13 inhibitors.

Two patients (14%) had transient ischemic attacks: a 72‐year‐old man with a *C3* variant (patient 9) and a 23‐year‐old woman with a *CFB* variant and no preexisting cardiovascular risk factors (patient 8). Further neurological symptoms were reported in the aHUS group: five (36%) of 14 patients reported a combination of headache (*n* = 4), visual disturbance (*n* = 1) and confusion (*n* = 1). Only one aHUS patient required intubation and ventilation at presentation due to cardiorespiratory failure.

Atypical hemolytic uremic syndrome precipitants at presentation or relapse included pregnancy (*n* = 2), upper respiratory tract infection (*n* = 6), vaccination (*n* = 2), and acute abdominal pathology (*n* = 3), with a case of appendicitis, pancreatitis, and acute non‐infective colitis. In three cases, there was no identifiable trigger (Table 1). Three patients had diarrhea at presentation; all were enterotoxin negative. One patient (patient 1) had a subsequent episode of enterotoxin‐positive *Escherichia coli* diarrhea as a precipitant for relapse. The pregnancy‐associated aHUS cases presented 3 days post cesarean section due to an abnormal cardiotocogram (patient 10) and 5 days after cesarean section at 31 weeks for presumed preeclampsia with nephrotic range proteinuria and a concurrent *Pseudomonas* bacteremia (patient 7). Subsequent identification of a pathogenic *CFH* mutation confirmed the diagnosis of aHUS in the latter case.

### Complement analysis

C3 and C4 levels were both reduced in three (27%) of 11 patients tested during an acute presentation. In 64% (nine of 14 patients), rare genetic variants in the alternative complement pathway were identified: five *CD46* (one compound heterozygote, four heterozygotes [two *CFH*, one *CFB*, and one *C3*]. The variants identified and complement antigenic levels are detailed in Table 2.

Mutation screening in one individual (patient 1) showed a compound heterozygote *CD46* mutation (c.286+2T>C, c.286+2T>G), which resulted in complete CD46 deficiency. The c.286+2T>C variant was of maternal origin, while the c.286+2T>G was paternal. Flow cytometry analysis confirmed absent CD46 expressed on peripheral blood mononuclear cells. Both parents had 50% CD46 expression with normal serum creatinine. The CD46 variants Arg59X 27, 28 and Cys64Phe 29 have been reported previously and result in impaired expression. CD46 expression data on the individuals carrying the Cys157Tyr genetic variant are not available; however, the loss of a structurally critical cysteine is highly likely to result in a non‐secreted protein.

The *CFH* variant p.Arg1215X (patient 7) has been reported 1 and results in impaired protein secretion. Analysis of cDNA from the patient with the *CFH* c.3134‐5T>C genetic variant (patient 6) did not detect any splice products that differed from the expected transcript sequence seen in normal control samples. The FH serum level was normal. A Western blot using a polyclonal antibody against FH did not detect any aberrant FH species (Fig. S1). Thus, from analysis undertaken to date, we cannot ascribe a functional consequence to this variant.

**Figure 2:**
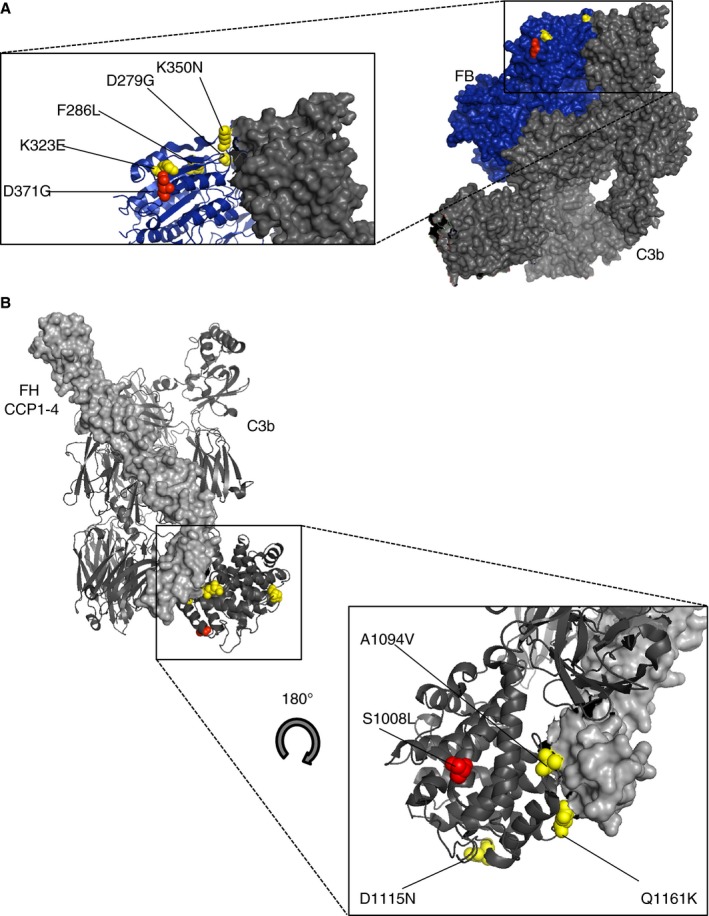
Location of novel *CFB* and *C3* variants described in this study. (A) The *CFB* genetic variant Asp371Gly displayed (red sphere) on the C3b (dark gray): FB (blue) co‐crystal structure (Protein Data Base ID code 2XWJ) 53. Previously reported functionally significant atypical hemolytic uremic syndrome (aHUS)‐associated *CFB* genetic variants Asp279Gly, Phe286Leu, Lys323Glu, and Lys350Asn (yellow spheres) are shown residing in the von Willebrand type A domain, inset 54. (B) An X‐ray–derived co‐crystal structure of FH/C3b was also used to model the genetic variant in *C3*. The location of the Ser1008Leu C3 variant (red sphere) is shown within the co‐crystal structure of an FH CCPs1–4 (light gray):C3b (dark gray) complex (Protein Data Base ID code 2WII) 55. Previously reported functionally significant aHUS‐associated *C3* genetic variants Ala1094Val, Asp1115Asn, and Gln1161Lys (yellow spheres) are shown inset 54.

The *CFB* variant p.Asp371Gly (patient 8) has not previously been described and is not reported to be present in the general population from public mutation databases (NHLBI GO ESP [National Heart, Lung, and Blood Institute Grand Opportunity Exome Sequencing Project]) 30. Polymorphism phenotyping v2 (PolyPhen‐2) 31 predicted that this variant is benign; however, previous *in silico* analysis of *CFB* mutants has demonstrated poor correlation with functional studies 32. Structural analysis using an available crystal structure of complement C3b in complex with FB demonstrates that this variant resides in the von Willebrand factor domain of FB 32 in close proximity to four other aHUS variants where functional analysis has demonstrated increased activity (Fig. 2A).

The C3 variant p. Ser1008Lys (patient 9) has not previously been described and is not reported in the NHLBI GO ESP 30. PolyPhen‐2 predicted that this variant is probably damaging, although bioinformatic evaluation should be taken with caution. A co‐crystal structure of CCPs1–4 of FH and C3b demonstrates that the variant resides in the thioester‐containing domain adjacent to three other functionally significant aHUS‐associated mutations Ala1094Val, Asp1115Asn, and Gln1161Lys 25 (Fig. 2B).

No patients with FH autoantibody were identified, although most were screened after TPE or while in clinical remission. At‐risk *CFH‐H3* or *CD46*GGAAC haplotypes were identified in all 14 patients tested (Table 2).

### Treatment and outcomes

Thirteen of 14 aHUS patients were treated initially with TPE; one patient (patient 13) had resolving TMA at the time of transfer and did not require treatment. One patient (patient 14) developed multiorgan failure and died within 24 h of presentation after receiving corticosteroids and one TPE. A post mortem study was not performed. Three patients required renal replacement therapy (RRT) during episodes of TMA with either peritoneal or hemodialysis on a total of five occasions (two at relapse) for a duration of between 4 days and 18 months. Median time from presentation to recovery of platelet count ≥ 150 × 10^9^ L^−1^ was 9 days (range 4–29 days). Only one patient, with a *CFB* variant (patient 8), had thrombocytopenia refractory to TPE, defined by lack of platelet response to 1 week of TPE, and received eculizumab 13 days after presentation at a dosage of 900 mg weekly for 3 weeks, with complete response in both thrombocytopenia and renal function. Eculizumab was not available for any other patient in this study at presentation or relapse.

Median follow‐up for the aHUS cohort was 2 years (range 1–28 years). Five of 13 patients had recurrent episodes; all had confirmed complement pathway genetic abnormalities (three *CD46*, one *CFB*, one *C3*). Two of the relapses occurred within 1 year of the first episode (range, 1 month to 8 years after index presentation) and primarily triggered by viral/bacterial infection or vaccinations (Table 1). The precipitant was unclear in one case (patient 9), who relapsed just over 1 month after discharge with his initial presentation. The patient with a *CFB* mutation (patient 8) relapsed > 1 year after achieving remission, precipitated by both vaccination and viral infection, and required an additional two doses of eculizumab. Of interest, she had an uncomplicated pregnancy between presentations, for which she did not receive eculizumab.

At most recent clinical review, all aHUS patients had platelet counts above 150 × 10^9^ L^−1^, and 12 of 13 had normal serum creatinine levels; one patient (patient 6) had stage 3 chronic kidney disease with a serum creatinine level of 122 μmol L^−1^. Two of 12 patients had persistent proteinuria (one with chronic renal impairment and one with disease recurrence), and four of 12 patients required treatment for persistent hypertension. None required long‐term RRT or progressed to ESRF.

### Complement analysis in TTP patients

Fourteen TTP patients underwent complement mutation analysis. The median platelet count was 13 × 10^9^ L^−1^ (range 5–74), and serum creatinine level was 106.5 μmol L^−1^ (61–353). Selection according to severe phenotypic presentation was based on a combination of clinical features. Four patients (29%) required intubation and ventilation at presentation, and two patients had a stroke. Four patients (29%) had marked renal impairment on presentation with creatinine > 150 μmol L^−1^. Five patients (36%) had thrombocytopenia refractory to 7 days of TPE. Six (43%) had experienced recurrent relapses (median of three acute presentations, range 2–6). All had ADAMTS‐13 activity below the lowest level of quantification (< 5%) with detectable ADAMTS‐13 inhibitors. No rare genetic variants were identified in *C3, CFB, CFI, CFH*, or *CD46* in this cohort.

## Discussion

Many studies have shown that TMA patients without ADAMTS‐13 deficiency have significantly higher median creatinine levels and platelet counts at presentation than those with confirmed acquired TTP 9, 33, 34. Although our findings were consistent with this, neither of these laboratory parameters demonstrated adequate specificity for aHUS, in a cohort of patients of whom most did not present with oliguric/anuric renal failure. The majority (13 of 14) of our aHUS patients would be misdiagnosed based on clinical criteria for differentiation of TTP from other TMAs suggested in previous publications 4, 33. Coppo *et al*. reported that either a platelet count ≤ 30 × 10^9^ L^−1^, creatinine level of ≤ 200 μmol L^−1^, or positive ANA has a 85% positive predictive value and 93.3% negative predictive value for severe ADAMTS‐13 deficiency in a large TTP/TMA cohort. Using only platelet count and serum creatinine, however, six of 54 patients (11%) with ADAMTS‐13 activity > 10% were incorrectly predicted to have ADAMTS‐13 deficiency based on these clinical variables alone. The intention of this study, which focused on diagnosis of TTP rather than aHUS, was not to define TMA classification according to clinical presentation, and the authors emphasize the need for pretreatment assessment of ADAMTS‐13 activity 33. Although the aHUS cohort presented here represents a selected group of patients, referred with suspected TTP, 13 of our 14 aHUS patients would be predicted to have severe ADAMTS‐13 deficiency. It is evident that patients with both TTP and aHUS present with laboratory values outside expected limits; therefore, ADAMTS‐13 testing is important for diagnosis and subsequent therapy.

Screening for complement abnormalities in TMA patients with renal impairment provides valuable diagnostic and prognostic information, although ~ 50% of patients have no detectable abnormalities 6. Diagnosis in these cases remains reliant on clinical features. Published studies on aHUS in the acute TMA setting often define this group by parameters such as serum creatinine 35. There are no established assays to measure global alternative complement pathway activity. In a search for potential biomarkers, C5a levels were recently shown to be significantly higher in aHUS than TTP, but there was significant overlap and no clear cut‐off could be suggested as a diagnostic tool 35. C3 or C4 levels are not reliable predictors, confirmed in our aHUS cohort.

In the absence of a rapidly available diagnostic test or biomarker for aHUS, the presence of ADAMTS‐13 deficiency has an important differentiating role in acute TMAs. In the majority of TTP studies, patients with ADAMTS‐13 activity > 10% had a significantly higher incidence and severity of renal impairment, a proportion of whom, in the absence of an identified secondary cause, may have had aHUS 36. Many predated knowledge of alternative complement pathway abnormalities in aHUS, and none included routine screening for complement mutations in those without severe ADAMTS‐13 deficiency. Cataland *et al*. reported that ADAMTS‐13 levels < 10% were 100% sensitive and 100% specific for TTP in a cohort of 57 patients compared with 57 TMA patients with alternative diagnoses 10. It has also been demonstrated that using an ADAMTS‐13 activity cut‐off of 10% can be safely used to guide TTP therapy 9.

Sixty‐four percent of our patients diagnosed with aHUS had variants in complement genes, consistent with a prevalence of 44–51% in larger cohorts 5, 6, 7. Given that complement analysis was performed largely on convalescent or post‐TPE samples, the presence of FH autoantibodies may potentially have been missed. Analysis for thrombomodulin 5, 37 and diacylglycerol kinase, epsilon (*DGKE*) 38 mutations was not undertaken. *DGKE* mutations are primarily detected in childhood, however, and all those presenting at a young age had mutations identified.

We identified a much higher frequency of isolated *CD46* variants (36% vs. 5–13%) and lower incidence of *CFH* variants (14% vs. 20–30%) than were reported in other studies 5, 6, 7, 27. Patients with isolated *CD46* mutations are known to have a milder clinical phenotype with reduced incidence of progression to ESRF (6–19%) 5, 6, 27. The higher proportion of *CD46* variants may account for the better outcomes in our aHUS cohort with reduced severity of renal impairment and favorable long‐term outcomes, compared with a 60% incidence of ESRF or death at 3 years in the largest aHUS cohort 6. CD46 is a membrane‐bound protein where the role of TPE in treatment is questionable and the majority of patients with isolated *CD46* variants have complete resolution of disease with or without TPE 5, 27.

We identified two *CD46*‐mutated patients (patients 3 and 4), presenting with a relapsing/remitting TMA clinically indistinguishable from TTP, with normal renal function and ADAMTS‐13 levels. Preceding the availability of ADAMTS‐13 assays, both were diagnosed with congenital TTP, based on their young age of presentation and relapsing episodes. In similar cases, confirmation of normal ADAMTS‐13 activity should trigger a thorough assessment for alternative causes of TMA, including complement mutation analysis. Future studies should redefine the normal ADAMTS‐13 ‘TTP’ group and incorporate molecular diagnostics to a greater extent.

Of particular interest is patient 1 with compound heterozygous deficiency of CD46. Currently, < 10 patients have been shown to have complete CD46 deficiency. It is reported that 50% of those patients have common variable immunodeficiency and recurrent chest infections. It has been suggested that impaired *CD46‐Jagged1* crosstalk is responsible for the recurrent infections in subpopulations of these patients 39. Our patient had recurrent upper respiratory tract infections and three episodes of reported chickenpox in childhood but has not experienced recurrent infections throughout adulthood.

All except one patient in this cohort received TPE as the primary treatment with positive clinical outcomes overall, before eculizumab was available nationally. With the confirmation of a pathogenic mutation, however, there is now a precedence to initiate eculizumab at relapse of an aHUS episode 4, 12. In our cohort, where only one individual received short intermittent courses of eculizumab, the median time to normalization of platelet count was 9 days and all except one patient (stage 3 chronic kidney disease) had complete recovery of renal function. A brief reintroduction of TPE or eculizumab (in one case) controlled relapsed disease in all patients. The duration of terminal complement blockade needs further clarification with reports suggesting eculizumab may be safely discontinued in selected patients with appropriate clinical monitoring 40, 41, a finding mirrored by our own experience.

Activation of the classical and alternative complement pathways in TTP has been demonstrated in several studies 42, 43, 44, although the relative contribution of complement to disease pathogenesis has not been fully elucidated. The TTP cohort presented here represented an exploratory group selected due to a severe clinical phenotype but was not intended for direct comparison with aHUS patients based on clinical parameters. We did not identify any congenital or acquired aberrations of the alternative complement pathway in this select group, suggesting the presence of dual pathologies is exceptional and that routine screening for complement mutations in TTP patients with ADAMTS‐13 levels < 10% and transient severe renal impairment is not necessary. It should be noted, however, that only four TTP patients in this study presented with creatinine levels > 150 μmol L^−1^. Larger‐scale studies would be required to further validate these findings.

## Conclusion

This aHUS cohort demonstrates the difficulty in clinically differentiating TTP from complement mediated TMAs. We highlight that diagnostic differentiation based on platelet count and renal function was insufficient to predict an underlying complement mutation. This distinction is increasingly important with the proven efficacy of complement inhibitor therapy in targeting complement activation in aHUS. In any patient with acute presentation of idiopathic TMA, identification of an ADAMTS‐13 activity > 10% without detectable anti–ADAMTS‐13 autoantibodies necessitates consideration of aHUS. Specifically, we demonstrate a higher frequency of functionally significant *CD46* mutations, closely mimicking relapsing/remitting TTP. We have illustrated that remissions can be associated with TPE in less severe phenotypic presentations, although with the availability of eculizumab, patient management is evolving. In a subgroup of patients with aHUS, continuous therapy is not required to maintain disease remission.

## Addendum

E. H. Phillips analyzed clinical data and wrote the manuscript. V. Brocklebank and J. O. Tellez performed genetic analysis and interpretation. E. K. S. Wong performed Western blotting. K. J. Marchbank performed FH autoantibody assays. S. McGuckin, D. P. Gale, J. Connolly, and T. H. J. Goodship presented clinical information and reviewed the final manuscript. D. Kavanagh undertook genetic analysis and modeling of genetic variants, designed the experiments, and contributed to the writing and reviewing of the manuscript. M. A. Scully designed the experiments, reviewed data, and contributed to the writing and reviewing of the manuscript. All authors approved the final version of the manuscript.

## Disclosure of Conflict of Interests

M. A. Scully, D. Kavanagh, T. H. J. Goodship, and E. K. S. Wong report personal fees and/or non‐financial support from Alexion Pharmaceuticals, outside the submitted work. The other authors state that they have no conflict of interest.

## Supporting information


**Fig. S1.** FH analysis from the patient with a *CFH* c.3134‐5T>C genetic variant. Western blot using a polyclonal antibody against FH demonstrates the absence of aberrant FH species. The FH concentration from the patient with the *CFH* c.3134‐5T>C is > 2× the control sample in an attempt to elucidate aberrant species.

## References

[1] Fremeaux‐Bacchi V , Fakhouri F , Garnier A , Bienaimé F , Dragon‐Durey M‐A , Ngo S , Moulin B , Servais A , Provot F , Rostaing L , Burtey S , Niaudet P , Deschênes G , Lebranchu Y , Zuber J , Loirat C . Genetics and outcome of atypical hemolytic uremic syndrome: a nationwide French series comparing children and adults. Clin J Am Soc Nephrol 2013; 8: 554–62. 23307876 10.2215/CJN.04760512 PMC3613948

[2] Noris M , Remuzzi G . Atypical hemolytic‐uremic syndrome. N Engl J Med 2009; 361: 1676–87. 19846853 10.1056/NEJMra0902814

[3] Hosler GA , Cusumano AM , Hutchins GM . Thrombotic thrombocytopenic purpura and hemolytic uremic syndrome are distinct pathologic entities: a review of 56 autopsy cases. Arch Pathol Lab Med 2003; 127: 834–9. 12823037 10.5858/2003-127-834-TTPAHU

[4] Zuber J , Fakhouri F , Roumenina LT , Loirat C , Frémeaux‐Bacchi V . Use of eculizumab for atypical haemolytic uraemic syndrome and C3 glomerulopathies. Nat Rev Nephrol 2012; 8: 643–57. 23026949 10.1038/nrneph.2012.214

[5] Noris M , Caprioli J , Bresin E , Mossali C , Pianetti G , Gamba S , Daina E , Fenili C , Castelletti F , Sorosina A , Piras R , Donadelli R , Maranta R , van der Meer I , Conway EM , Zipfel PF , Goodship TH , Remuzzi G . Relative role of genetic complement abnormalities in sporadic and familial aHUS and their impact on clinical phenotype. Clin J Am Soc Nephrol 2010; 5: 1844–59. 20595690 10.2215/CJN.02210310 PMC2974386

[6] Bresin E , Rurali E , Caprioli J , Sanchez‐Corral P , Fremeaux‐Bacchi V , Rodriguez de Cordoba S , Pinto S , Goodship THJ , Alberti M , Ribes D , Valoti E , Remuzzi G , Noris M . Combined complement gene mutations in atypical hemolytic uremic syndrome influence clinical phenotype. J Am Soc Nephrol 2013; 24: 475–86. 23431077 10.1681/ASN.2012090884 PMC3582207

[7] Maga TK , Nishimura CJ , Weaver AE , Frees KL , Smith RJH . Mutations in alternative pathway complement proteins in American patients with atypical hemolytic uremic syndrome. Hum Mutat 2010; 31: 2169–81. 10.1002/humu.21256 20513133

[8] Furlan M , Robles R , Galbusera M , Remuzzi G , Kyrle PA , Brenner B , Krause M , Scharrer I , Aumann V , Mittler U , Solenthaler M , Lämmle B . von Willebrand factor‐cleaving protease in thrombotic thrombocytopenic purpura and the hemolytic‐uremic syndrome. N Engl J Med 1998; 339: 1578–84. 9828245 10.1056/NEJM199811263392202

[9] Shah N , Rutherford C , Matevosyan K , Shen YM , Sarode R . Role of ADAMTS‐13 in the management of thrombotic microangiopathies including thrombotic thrombocytopenic purpura (TTP). Br J Haematol 2013; 163: 514–9. 24111495 10.1111/bjh.12569

[10] Cataland SR , Wu HM . How I treat: the clinical differentiation and initial treatment of adult patients with atypical hemolytic uremic syndrome. Blood 2014; 123: 2478–84. 24599547 10.1182/blood-2013-11-516237

[11] Scully M , McDonald V , Cavenagh J , Hunt BJ , Longair I , Cohen H , Machin SJ . A phase 2 study of the safety and efficacy of rituximab with plasma exchange in acute acquired thrombotic thrombocytopenic purpura. Blood 2011; 118: 1746–53. 21636861 10.1182/blood-2011-03-341131

[12] Legendre CM , Licht C , Muus P , Greenbaum L a , Babu S , Bedrosian C , Bingham C , Cohen DJ , Delmas Y , Douglas K , Eitner F , Feldkamp T , Fouque D , Furman RR , Gaber O , Herthelius M , Hourmant M , Karpman D , Lebranchu Y , Mariat C , *et al* Terminal complement inhibitor eculizumab in atypical hemolytic‐uremic syndrome. N Engl J Med 2013; 368: 2169–81. 23738544 10.1056/NEJMoa1208981

[13] Scully M , Hunt BJ , Benjamin S , Liesner R , Rose P , Peyvandi F , Cheung B , Machin SJ . Guidelines on the diagnosis and management of thrombotic thrombocytopenic purpura and other thrombotic microangiopathies. Br J Haematol 2012; 158: 323–35. 22624596 10.1111/j.1365-2141.2012.09167.x

[14] Haemolytic uraemic syndrome ‐ clinician information. version 5 Jan 2015. 2015 www.rarerenal.org/clinician. Accessed 23 December 2015.

[15] Ariceta G , Besbas N , Johnson S , Karpman D , Landau D , Licht C , Loirat C , Pecoraro C , Taylor CM , van der Kar N , VandeWalle J , Zimmerhackl LB . Guideline for the investigation and initial therapy of diarrhea‐negative hemolytic uremic syndrome. Pediatr Nephrol 2009; 24: 687–96. 18800230 10.1007/s00467-008-0964-1

[16] Kidney Disease: Improving Global Outcomes (KDIGO) Acute Kidney Injury Work Group . KDIGO Clinical Practice Guideline for Acute Kidney Injury. Kidney Int 2012; 2: 1–138.

[17] Kokame K , Nobe Y , Kokubo Y , Okayama A , Miyata T . FRETS‐VWF73, a first fluorogenic substrate for ADAMTS‐13 assay. Br J Haematol 2005; 129: 93–100. 15801961 10.1111/j.1365-2141.2005.05420.x

[18] Scully M , Yarranton H , Liesner R , Cavenagh J , Hunt B , Benjamin S , Bevan D , Mackie I , Machin S . Regional UK TTP registry: correlation with laboratory ADAMTS 13 analysis and clinical features. Br J Haematol 2008; 142: 819–26. 18637802 10.1111/j.1365-2141.2008.07276.x

[19] Moore I , Strain L , Pappworth I , Kavanagh D , Barlow P , Herbert A , Schmidt C , Staniforth S , Holmes L , Ward R , Morgan L , Goodship T , Marchbank K . Association of factor H autoantibodies with deletions of CFHR1, CFHR3, CFHR4, and with mutations in CFH, CFI, CD46, and C3 in patients with atypical hemolytic uremic syndrome. Blood 2010; 115: 379–87. 19861685 10.1182/blood-2009-05-221549 PMC2829859

[20] Brocklebank V , Wong EKS , Fielding R , Goodship THJ , Kavanagh D . Atypical haemolytic uraemic syndrome associated with a CD46 mutation triggered by Shigella flexneri. Clin Kidney J 2014; 7: 286–8. 24944786 10.1093/ckj/sfu032 PMC4038258

[21] Wong EKS , Anderson HE , Herbert AP , Challis RC , Brown P , Reis GS , Tellez JO , Strain L , Fluck N , Humphrey A , Macleod A , Richards A , Ahlert D , Santibanez‐Koref M , Barlow PN , Marchbank KJ , Harris CL , Goodship THJ , Kavanagh D . Characterization of a factor H mutation that perturbs the alternative pathway of complement in a family with membranoproliferative GN. J Am Soc Nephrol 2014; 25: 2425–33. 24722444 10.1681/ASN.2013070732 PMC4214516

[22] Kavanagh D . Mutations in complement factor I predispose to development of atypical hemolytic uremic syndrome. J Am Soc Nephrol 2005; 16: 2150–5. 15917334 10.1681/ASN.2005010103

[23] Kavanagh D , Kemp EJ , Richards A , Burgess RM , Mayland E , Goodship JA , Goodship THJ . Does complement factor B have a role in the pathogenesis of atypical HUS? Mol Immunol 2006; 43: 856–9. 16061287 10.1016/j.molimm.2005.06.041

[24] Richards A , Kemp EJ , Liszewski MK , Goodship JA , Lampe AK , Decorte R , Müslümanoğlu MH , Kavukcu S , Filler G , Pirson Y , Wen LS , Atkinson JP , Goodship THJ . Mutations in human complement regulator, membrane cofactor protein (CD46), predispose to development of familial hemolytic uremic syndrome. Proc Natl Acad Sci U S A 2003; 100: 12966–71. 14566051 10.1073/pnas.2135497100 PMC240728

[25] Frémeaux‐Bacchi V , Miller EC , Liszewski MK , Strain L , Blouin J , Brown AL , Moghal N , Kaplan BS , Weiss RA , Lhotta K , Kapur G , Mattoo T , Nivet H , Wong W , Gie S , De Ligny BH , Fischbach M , Gupta R , Hauhart R , Meunier V , *et al* Mutations in complement C3 predispose to development of atypical hemolytic uremic syndrome. Blood 2008; 112: 4948–52. 18796626 10.1182/blood-2008-01-133702 PMC2597601

[26] Francis NJ , McNicholas B , Awan A , Waldron M , Reddan D , Sadlier D , Kavanagh D , Strain L , Marchbank KJ , Harris CL , Goodship THJ . A novel hybrid CFH/CFHR3 gene generated by a microhomology‐mediated deletion in familial atypical hemolytic uremic syndrome. Blood 2012; 119: 591–601. 22058112 10.1182/blood-2011-03-339903

[27] Caprioli J , Noris M , Brioschi S , Pianetti G , Castelletti F , Bettinaglio P , Mele C , Bresin E , Cassis L , Gamba S , Porrati F , Bucchioni S , Monteferrante G , Fang CJ , Liszewski MK , Kavanagh D , Atkinson JP , Remuzzi G . Genetics of HUS: the impact of MCP, CFH, and IF mutations on clinical presentation, response to treatment, and outcome. Blood 2006; 108: 1267–79. 16621965 10.1182/blood-2005-10-007252 PMC1895874

[28] Fremeaux‐Bacchi V , Moulton EA , Kavanagh D , Dragon‐Durey M‐A , Blouin J , Caudy A , Arzouk N , Cleper R , Francois M , Guest G , Pourrat J , Seligman R , Fridman WH , Loirat C , Atkinson JP . Genetic and functional analyses of membrane cofactor protein (CD46) mutations in atypical hemolytic uremic syndrome. J Am Soc Nephrol 2006; 17: 2017–25. 16762990 10.1681/ASN.2005101051

[29] Kwon T , Belot A , Ranchin B , Baudouin V , Fremeaux‐Bacchi V , Dragon‐Durey MA , Cochat P , Loirat C . Varicella as a trigger of atypical haemolytic uraemic syndrome associated with complement dysfunction: two cases. Nephrol Dial Transplant 2009; 24: 2752–4. 19376828 10.1093/ndt/gfp166

[30] Tennessen JA , Bigham AW , O'Connor TD , Fu W , Kenny EE , Gravel S , McGee S , Do R , Liu X , Jun G , Kang HM , Jordan D , Leal SM , Gabriel S , Rieder MJ , Abecasis G , Altshuler D , Nickerson DA , Boerwinkle E , Sunyaev S , *et al* Evolution and functional impact of rare coding variation from deep sequencing of human exomes. Science 2012; 337: 64–9. 22604720 10.1126/science.1219240 PMC3708544

[31] Adzhubei IA , Schmidt S , Peshkin L , Ramensky VE , Gerasimova A , Bork P , Kondrashov AS , Sunyaev SR . A method and server for predicting damaging missense mutations. Nat Methods 2010; 7: 248–9. 20354512 10.1038/nmeth0410-248 PMC2855889

[32] Marinozzi M , Vergoz L , Rybkine T , Ngo S , Bettoni S , Pashov A , Cayla M , Tabarin F , Jablonski M , Hue C , Smith R , Noris M , Halbwachs‐Mecarelli L , Donadelli R , Fremeaux‐Bacchi V , Roumenina L . Complement factor B mutations in atypical hemolytic uremic syndrome‐disease‐relevant or benign? J Am Soc Nephrol 2014; 25: 2053–65. 24652797 10.1681/ASN.2013070796 PMC4147975

[33] Coppo P , Schwarzinger M , Buffet M , Wynckel A , Clabault K , Presne C , Poullin P , Malot S , Vanhille P , Azoulay E , Galicier L , Lemiale V , Mira JP , Ridel C , Rondeau E , Pourrat J , Girault S , Bordessoule D , Saheb S , Ramakers M , *et al* Predictive features of severe acquired ADAMTS‐13 deficiency in idiopathic thrombotic microangiopathies: the French TMA reference center experience. PLoS ONE 2010; 5: 1–9. 10.1371/journal.pone.0010208 PMC2859048 20436664

[34] Cataland SR , Yang S , Wu HM . The use of ADAMTS‐13 activity, platelet count and serum creatinine to differentiate acquired thrombotic thrombocytopenic purpura from other thrombotic microangiopathies. Br J Haematol [Internet] 2012; 157: 501–3. 10.1111/j.1365-2141.2012.09032.x 22296585

[35] Cataland SR , Holers V , Geyer S , Yang S , Wu HM . Biomarkers of the alternative pathway and terminal complement activity at presentation confirms the clinical diagnosis of aHUS and differentiates aHUS from TTP. Blood 2014; 123: 3733–8. 24695849 10.1182/blood-2013-12-547067

[36] Vesely SK , George JN , Lämmle B , Studt JD , Alberio L , El‐Harake MA , Raskob GE . ADAMTS‐13 activity in thrombotic thrombocytopenic purpura‐hemolytic uremic syndrome: relation to presenting features and clinical outcomes in a prospective cohort of 142 patients. Blood 2003; 102: 60–8. 12637323 10.1182/blood-2003-01-0193

[37] Delvaeye M , Noris M , De Vriese A , Esmon CT , Esmon NL , Ferrell G , Del‐Favero J , Plaisance S , Claes B , Lambrechts D , Zoja C , Remuzzi G , Conway EM . Thrombomodulin mutations in atypical hemolytic‐uremic syndrome. N Engl J Med 2009; 361: 345–57. 19625716 10.1056/NEJMoa0810739 PMC3530919

[38] Lemaire M , Frémeaux‐Bacchi V , Schaefer F , Choi M , Tang WH , Le Quintrec M , Fakhouri F , Taque S , Nobili F , Martinez F , Ji W , Overton JD , Mane SM , Nürnberg G , Altmüller J , Thiele H , Morin D , Deschenes G , Baudouin V , Llanas B , *et al* Recessive mutations in DGKE cause atypical hemolytic‐uremic syndrome. Nat Genet 2013; 45: 531–6. 23542698 10.1038/ng.2590 PMC3719402

[39] Le Friec G , Sheppard D , Whiteman P , Karsten CM , Al S , Shamoun T , Laing A , Bugeon L , Dallman MJ . The CD46 and Jagged1 interaction is critical for human T helper 1 immunity. Nat Immunol 2012; 13: 1213–21. 23086448 10.1038/ni.2454 PMC3505834

[40] Ardissino G , Testa S , Possenti I , Tel F , Paglialonga F , Salardi S , Tedeschi S , Belingheri M , Cugno M . Discontinuation of eculizumab maintenance treatment for atypical hemolytic uremic syndrome: a report of 10 cases. Am J Kidney Dis 2014; 64: 633–7. 24656451 10.1053/j.ajkd.2014.01.434

[41] Sheerin NS , Kavanagh D , Goodship THJ , Johnson S . A national specialized service in England for atypical haemolytic uraemic syndrome – the first year's experience. QJM 2015. pii: hcv082. 10.1093/qjmed/hcv082 25899302

[42] Ruiz‐Torres MP , Casiraghi F , Galbusera M , Macconi D , Gastoldi S , Todeschini M , Porrati F , Belotti D , Pogliani EM , Noris M , Remuzzi G . Complement activation: the missing link between ADAMTS‐13 deficiency and microvascular thrombosis of thrombotic microangiopathies. Thromb Haemost 2005; 93: 443–52. 15735793 10.1160/TH04-07-0450

[43] Réti M , Farkas P , Csuka D , Rázsó K , Schlammadinger Á , Udvardy ML , Madách K , Domján G , Bereczki C , Reusz GS , Szabó AJ , Prohászka Z . Complement activation in thrombotic thrombocytopenic purpura. J Thromb Haemost 2012; 10: 791–8. 22372946 10.1111/j.1538-7836.2012.04674.x

[44] Westwood J‐P , Langley K , Heelas E , Machin SJ , Scully M . Complement and cytokine response in acute Thrombotic Thrombocytopenic Purpura. Br J Haematol 2014; 164: 858–66. 24372446 10.1111/bjh.12707 PMC4155869

[45] Richards A , Kathryn Liszewski M , Kavanagh D , Fang CJ , Moulton E , Fremeaux‐Bacchi V , Remuzzi G , Noris M , Goodship THJ , Atkinson JP . Implications of the initial mutations in membrane cofactor protein (MCP; CD46) leading to atypical hemolytic uremic syndrome. Mol Immunol 2007; 44: 111–22. 16882452 10.1016/j.molimm.2006.07.004

[46] Sullivan M , Erlic Z , Hoffmann MM , Arbeiter K , Patzer L , Budde K , Hoppe B , Zeier M , Lhotta K , Rybicki LA , Bock A , Berisha G , Neumann HPH . Epidemiological approach to identifying genetic predispositions for atypical hemolytic uremic syndrome. Ann Hum Genet 2010; 74: 17–26. 20059470 10.1111/j.1469-1809.2009.00554.x

[47] Pickering MC , de Jorge EG , Martinez‐Barricarte R , Recalde S , Garcia‐Layana A , Rose KL , Moss J , Walport MJ , Cook HT , de Córdoba SR , Botto M . Spontaneous hemolytic uremic syndrome triggered by complement factor H lacking surface recognition domains. J Exp Med 2007; 204: 1249–56. 17517971 10.1084/jem.20070301 PMC2118613

[48] Esparza‐Gordillo J , Goicoechea de Jorge E , Buil A , Berges LC , López‐Trascasa M , Sánchez‐Corral P , Rodríguez de Córdoba S . Predisposition to atypical hemolytic uremic syndrome involves the concurrence of different susceptibility alleles in the regulators of complement activation gene cluster in 1q32. Hum Mol Genet 2005; 14: 703–12. 15661753 10.1093/hmg/ddi066

[49] Zipfel PF , Edey M , Heinen S , Józsi M , Richter H , Misselwitz J , Hoppe B , Routledge D , Strain L , Hughes AE , Goodship JA , Licht C , Goodship THJ , Skerka C . Deletion of complement factor H‐related genes CFHR1 and CFHR3 is associated with atypical hemolytic uremic syndrome. PLoS Genet 2007; 3: 387–92. 10.1371/journal.pgen.0030041 PMC1828695 17367211

[50] Józsi M , Licht C , Strobel S , Zipfel SLH , Richter H , Heinen S , Zipfel PF , Skerka C . Factor H autoantibodies in atypical hemolytic uremic syndrome correlate with CFHR1/CFHR3 deficiency. Blood 2008; 111: 1512–4. 18006700 10.1182/blood-2007-09-109876

[51] Dragon‐Durey M‐A , Sethi SK , Bagga A , Blanc C , Blouin J , Ranchin B , André J‐L , Takagi N , Cheong H Il , Hari P , Le Quintrec M , Niaudet P , Loirat C , Fridman WH , Frémeaux‐Bacchi V . Clinical features of anti‐factor H autoantibody‐associated hemolytic uremic syndrome. J Am Soc Nephrol 2010; 21: 2180–7. 21051740 10.1681/ASN.2010030315 PMC3014031

[52] Geerdink LM , Westra D , van Wijk JAE , Dorresteijn EM , Lilien MR , Davin JC , Kömhoff M , van Hoeck K , van der Vlugt A , van den Heuvel LP , van de Kar NCAJ . Atypical hemolytic uremic syndrome in children: complement mutations and clinical characteristics. Pediatr Nephrol 2012; 27: 1283–91. 22410797 10.1007/s00467-012-2131-y PMC3382652

[53] Forneris F , Ricklin D , Wu J , Tzekou A , Wallace RS , Lambris JD , Gros P . Structures of C3b in complex with factors B and D give insight into complement convertase formation. Science 2010; 330: 1816–20. 21205667 10.1126/science.1195821 PMC3087196

[54] Kavanagh D , Goodship TH , Richards A . Atypical hemolytic uremic syndrome. Semin Nephrol 2013; 33: 508–30. 24161037 10.1016/j.semnephrol.2013.08.003 PMC3863953

[55] Wu J , Wu Y‐Q , Ricklin D , Janssen BJC , Lambris JD , Gros P . Structure of complement fragment C3b‐factor H and implications for host protection by complement regulators. Nat Immunol 2009; 10: 728–33. 19503104 10.1038/ni.1755 PMC2713992

