# Supplementary figures and images for: The role of ADAMTS‐13 activity and complement mutational analysis in differentiating acute thrombotic microangiopathies
Source: J Thromb Haemost. 2016 Jan 11;14(1):175–85. doi: 10.1111/jth.13189 (PMC4737436; doi:10.1111/jth.13189)

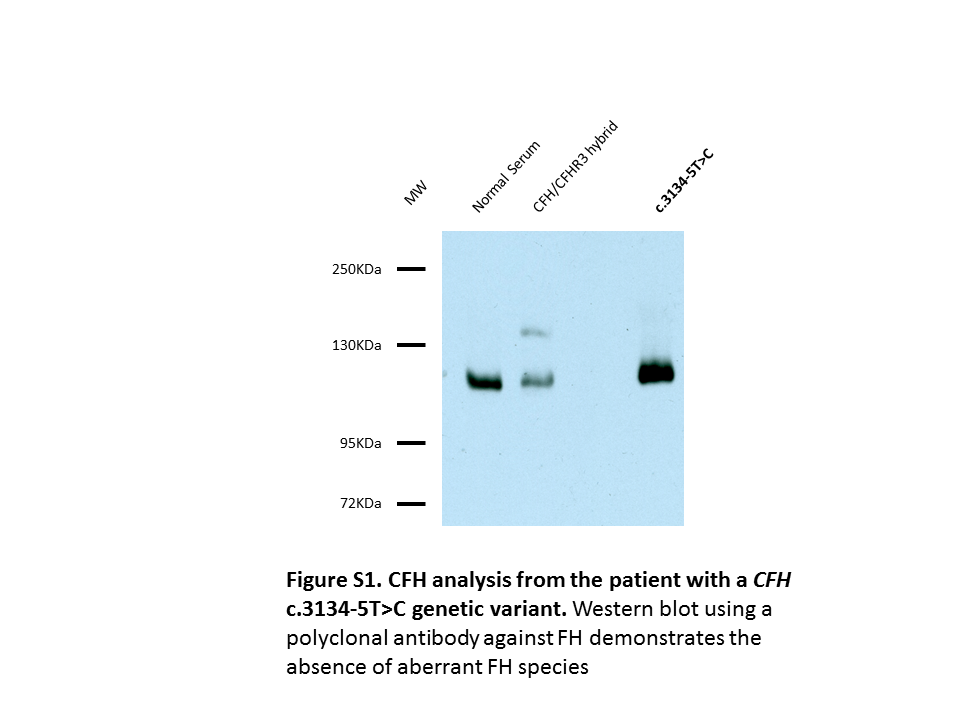

Supplement: Supplementary file 1 — Fig. S1. FH analysis from the patient with a CFH c.3134‐5T>C genetic variant. Western blot using a polyclonal antibody against FH demonstrates the absence of aberrant FH species. The FH concentration from the patient with the CFH c.3134‐5T>C is > 2× the control sample in an attempt to elucidate aberrant species. [file JTH-14-175-s001.tif]
